# Dietary *Aspergillus oryzae* Modulates Serum Biochemical Indices, Immune Responses, Oxidative Stress, and Transcription of *HSP70* and Cytokine Genes in Nile Tilapia Exposed to Salinity Stress

**DOI:** 10.3390/ani11061621

**Published:** 2021-05-31

**Authors:** Mustafa Shukry, Marwa F. Abd El-Kader, Basma M. Hendam, Mahmoud A. O. Dawood, Foad A. Farrag, Salama Mostafa Aboelenin, Mohamed Mohamed Soliman, Hany M. R. Abdel-Latif

**Affiliations:** 1 Department of Physiology, Faculty of Veterinary Medicine, Kafrelsheikh University, Kafrelsheikh 33516, Egypt; mostafa.ataa@vet.kfs.edu.eg; 2 Fish Diseases and Management, Sakha Aquaculture Research Unit, Central Laboratory for Aquaculture Research (A.R.C.), Cairo 11511, Egypt; m.abdelkader2000@yahoo.com; 3 Genetics and Genetic Engineering, Department of Husbandry and Development of Animal Wealth, Faculty of Veterinary Medicine, Mansoura University, Mansoura 35516, Egypt; d.basma.genetic@gmail.com; 4 Department of Animal Production, Faculty of Agriculture, Kafrelsheikh University, Kafrelsheikh 33516, Egypt; mahmoud.dawood@agri.kfs.edu.eg; 5 Department of Anatomy and Embryology, Faculty of Veterinary Medicine, Kafrelsheikh University, Kafrelsheikh 33516, Egypt; foad.farrag@vet.kfs.edu.eg; 6 Biology Department, Turabah University College, Taif University, P.O. Box 11099, Taif 21944, Saudi Arabia; s.aboelenin@tu.edu.sa; 7 Clinical Laboratory Sciences Department, Turabah University College, Taif University, P.O. Box 11099, Taif 21944, Saudi Arabia; mmsoliman@tu.edu.sa; 8 Department of Poultry and Fish Diseases, Faculty of Veterinary Medicine, Alexandria University, Alexandria 22758, Egypt

**Keywords:** probiotics, antioxidants, stress attenuation, non-specific immunity, qPCR

## Abstract

**Simple Summary:**

Probiotics are live microbial adjuncts with numerous beneficial effects on fish. This study aims to evaluate the roles of *Aspergillus oryzae* (ASP) in the modulation of serum haemato-biochemical measurements, immunity, antioxidative capacity, and transcriptomic responses of Nile tilapia juveniles exposed to salinity stress. Findings revealed that dietary supplementation with *A. oryzae* mitigated the harmful influences of salinity stress on the exposed Nile tilapia.

**Abstract:**

Nile tilapia Juveniles (19.50 ± 0.5 g) were fed on a basal diet (control group (CTR)) and a diet supplemented with 1 g *Aspergillus oryzae* (ASP) per kg diet for 12 weeks. Fish were then subjected to different salinity levels (0, 10, 15, and 20 practical salinity units (psu)) for another 15 days. Two-way ANOVA analysis revealed that the individual effects of ASP in Nile tilapia exposed to salinity levels presented a significant decrease (*p* < 0.05) in values of haemato-biochemical indices (such as glucose, cortisol, alanine transaminase, aspartate transaminase, and malondialdehyde) compared to those in the CTR group exposed to the same salinity levels. Moreover, significant increases (*p* < 0.05) of blood protein profile (albumin, globulin, and total protein), non-specific immune responses (lysozyme activity, phagocytic activity, and phagocytic index), and antioxidant enzymes activities (glutathione peroxidase, catalase, and superoxide dismutase) were observed in ASP-supplemented groups. Interestingly, there was significant (*p* < 0.05) downregulation of the mRNA expression values of heat shock protein 70 and interferon-gamma genes, alongside upregulation of the mRNA expression values of interleukin 1 beta and interleukin 8 genes, in the hepatic tissues of Nile tilapia in ASP-supplemented groups exposed to different salinities compared to those in the CTR group exposed to the same salinity levels. Taken together, these findings supported the potential efficacy of dietary supplementation with ASP in alleviating salinity stress-induced haemato-biochemical alterations, immune suppression, and oxidative stress in the exposed Nile tilapia.

## 1. Introduction

Over the past decade, the production rates of Nile tilapia (*Oreochromis niloticus*) have been greatly increased because of increased consumer preferences, relatively reasonable prices, and to some extent, its resistance to diseases [1]. During intensive culture systems, Nile tilapia are exposed to several stressors, which will weaken the fish immune responses and become susceptible to diseases [2]. The overuse of antibiotics for controlling disease occurrence has several drawbacks (for instance, the existence of multiple resistant bacteria harmfully affects the fish health and the persistence of drug residues in the tissues of treated fish), which will negatively affect human health [3]. Therefore, finding safe and environmentally friendly alternatives to improve fish health and productivity is of great interest to several researchers [4,5].

Probiotics are live microbial adjuncts that present several beneficial effects to a host, such as improving feed efficiency, enhancing growth performance, and fortifying the host immunity of fish against challenging environmental conditions [6]. The proficiency of probiotics to promote the health status is closely linked to their capability to augment the fish immunity and prevent the growth of pathogenic bacteria [7]. *Aspergillus oryzae* (ASP), as a probiotic, has been recognized as a significant source of various enzymes such as protease, glucoamylase, and alpha-amylase [8], and could be used as a probiotic feed supplement for poultry and livestock [9]. In Nile tilapia, dietary supplementation with *A. oryzae* could potentially enhance the immune responses and disease resistance [10], growth performance indices, intestinal histomorphometry [11], and antioxidative capacity [12], and improve defensive mechanisms against hypoxia stress [13]. Moreover, it enhances the growth and hematological parameters of common carp [14].

Salinity is one of the environmental factors that negatively affect the growth and reproduction performance of fish [15]. Salinity stress negatively influences the metabolic responses of shortnose sturgeon (*Acipenser brevirostrum*) juveniles [16] and the hematological indices of great sturgeon (*Huso huso*) [17]. Recently, Tian et al. [18] illuminated that exposure of the yellow drum (*Nibea albiflora*) to high salinities induced growth suppression, alterations of the intestinal microbiome, and changes in the physiological and immune-related parameters. It has been found that exposure of tilapia spp. to long-term hypersaline stress deleteriously induced several side effects such as impairment of reproductive performance [19], low survival rates [20], poor growth performance and impaired antioxidant capacity [21], histopathological alterations of spleen and kidneys [22], and disruption of the gut microbiome [23].

Cytokines are signaling molecules generated by immune cells to promote inflammation at the infection site and subsequently enhance the arrival of phagocytic cells to engulf and kill the invading pathogenic agents [24]. Moreover, Heat shock protein 70 (*HSP70*) is an indicator of stress in aquatic animals [25]. Some previously published studies illustrated that exposure of animals to chemicals and stress factors will induce liver injury [26,27]. In a similar sense, the mRNA expression levels of cytokines and *HSP70* were increased in the livers of Nile tilapia exposed to stressors and toxicants [28,29].

Several attempts have been made using dietary supplements to alleviate the effects of salinity stress, such as galacto-oligosaccharide [30], L-Tryptophan [30,31,32], *Lactobacillus rhamnosus* [33], taurine [34], bovine lactoferrin [35], mannan oligosaccharides [36], inulin [37], a host-derived *Bacillus subtilis* [38], and myo-inositol [39]. Hence, the current study aimed to evaluate the mitigative effect of *A. oryzae* on salinity stress through an exploration of the non-specific immunity, haemato-biochemical indices, antioxidative capacity, as well as the transcriptomic profile of HSP70 and cytokine genes in Nile tilapia.

## 2. Materials and Methods

### 2.1. Ethical Approval

The guidelines of the Local Experimental Animal Care Committee were closely followed during the present study. Moreover, this study was also approved by the Institutional Ethics Committee of the Faculty of Veterinary Medicine, Kafrelsheikh University (Approval No. 70 20-03-2018).

### 2.2. Probiotic Feed Supplement

*Aspergillus oryzae* (ASP) (Bio’c Co., Ltd., Uchida, Japan) contained 1 × 10^8^ CFU (colony forming units) per 1 g and was used as a feed supplement in the current study.

### 2.3. Fish Rearing and Management

Nile tilapia (*Oreochromis niloticus*) (19.50 ± 0.5 g) were procured from a local fish farm (Kafrelsheikh, Egypt) and then kept for 14 days in 500 L tanks, in the wet laboratory that belonged to Sakha Aquaculture Research Unit (Central Laboratory for Aquaculture Research, Kafrelsheikh, Egypt), to be acclimated to the laboratory conditions. During acclimation, fish were fed on a commercially purchased basal diet that was used as the control (CTR) diet (30% protein) (Aller Aqua Co., Giza, 3rd. Giza Governorate, Egypt). Ration ingredients and their chemical compositions were adapted to assemble the nutritional requirements for optimum growth of Nile tilapia (Table S1—please see Supplementary Materials). During the experimental periods, fish were reared in glass aquaria contained 100 L water and sized 1 × 1 × 1 m and supplemented with compressed air via air stones using aquarium air pumps. One third of the water per each aquarium was siphoned every 2 or 3 days to get rid of faecal matter and then substituted with new water from the storage tanks.

### 2.4. Water Quality Parameters

The physical and chemical parameters of the rearing water samples were examined weekly and maintained throughout the whole experimental period at 27.0–28.5 °C, 7.50 ± 0.50 mg/L, 7.5–8.5, and 0.01 ± 0.03 mg/L for temperature, dissolved oxygen, pH, and un-ionized ammonia, respectively [40].

### 2.5. Experimental Setup

#### 2.5.1. Experiment I: Feeding Trial

**Figure 1:**
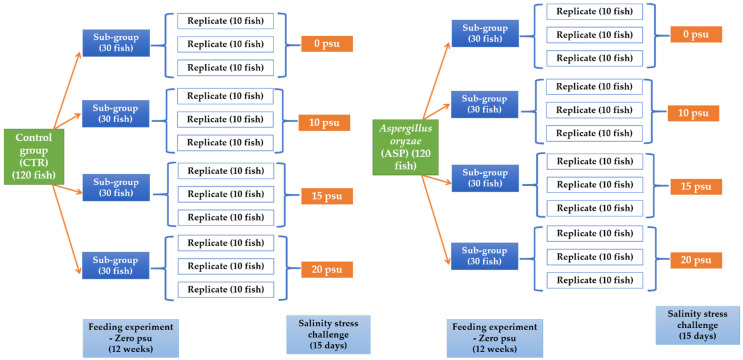
Experimental design of the present study showing the two experiments (feeding trial and salinity stress challenge).

A total number of 240 fish were allocated into two main groups (each group was divided into 4 sub-groups) (30 fish per each sub-group) (Figure 1). Fish were fed on two experimental diets that were prepared to contain two levels of *A. oryzae* (ASP) (0 g per kg diet) and served as a control group (CTR) (the commercially purchased diet), and the other group was fed the CTR diet supplemented with 1 g *A. oryzae* (ASP) per kg. ASP level used in the current study was selected according to our previously published studies on Nile tilapia [11,12,13]. Sodium carboxymethyl cellulose was used for pelleting the experimental diets (to make the diets as pellets to fit the fish feeding). Pellets were produced by an extruder pelleting machine and then kept in polyethylene bags at 4 °C until use. The experiment lasted for 12 weeks, and fish were fed daily until apparent satiety (3 times daily). During experiment I, fish were reared on freshwater with zero practical salinity units (psu).

#### 2.5.2. Experiment II: Challenge with Salinity Stress

After the end of the first experiment, the fish in each sub-group (30 fish) were allocated into 3 other replicates (10 fish per each) (Figure 1). Moreover, the fish continued to be fed on the same previously prepared and corresponding experimental diets (ASP and CTR). Afterward, the fish in each sub-group were exposed to a different salinity level (0, 10, 15, or 20 psu) for an additional 15 days (Figure 1).

During the salinity stress test, the fish in each glass aquarium were provided with continuous aeration. For the salinity challenge, the tap water was dechlorinated by strong aeration for 24 h and then mixed with seawater to raise the salinity to 10, 15, and 20 psu. Salinity concentrations were determined by a salinometer (Thermo Electron corporation model Orion 150 A+). After exposure to salinity levels, mortalities were recorded, and blood and tissue specimens were sampled.

### 2.6. Sampling

#### 2.6.1. Blood Sampling

Fish in all experimental groups were starved for 24 h before collection of blood samples. Three fish were sampled from each replicate (9 fish per sub-group). Fish were anesthetized by tricaine methane sulphonate, then blood was sampled from the caudal veins. Blood was allotted into two sets: one was collected with heparin for hematological parameters; the second set was left at room temperature (for serum collection into centrifuge tubes). Centrifugation was conducted at 3000× *g* for 15 min to separate serum, which were then stored at −20 °C until use.

#### 2.6.2. Liver Samples

Three liver samples from each replicate (9 samples per sub-group) were sampled and then immediately stored in liquid nitrogen and stored at −80 °C in TRIzol reagent (iNtRON Biotechnology, Inc., Gyeonggi, Korea) to be employed for PCR assays.

### 2.7. Serum Biochemical Measurements

Serum samples were investigated to obtain their protein profiles, which include total protein (TP) [41] and albumin (ALB) contents [42]. Globulin (GLO) levels were also determined by subtraction of ALB from TP values. Blood glucose (GLU), cortisol (CORT) levels, serum alanine transaminase (ALT) and aspartate transaminase (AST) activities were measured colorimetrically using commercially purchased specific kits (Bio diagnostic Co., Giza, Egypt) (all laboratory procedures were accomplished according to the protocol provided from the manufacturer).

### 2.8. Immunity and Antioxidant Parameters

#### 2.8.1. Immune Parameters

Serum lysozyme (LYZ) activity was evaluated by the turbidimetric assay using lyophilized *Micrococcus lysodeikticus* cells (Sigma-Aldrich, St. Louis, MN, USA) according to the methodology described by Ellis [43] and Mörsky [44].

Phagocytic activity (PA) and phagocytic index (PI) were calculated in the whole blood samples following the methodology of Kawahara et al. [45] according to the following equations:

1. PA = Total No. of macrophages encompassing yeast cells/total No. of macrophages × 100
2. PI = No. of phagocytized yeast cells/No. of phagocytic cells

#### 2.8.2. Oxidative Stress Biomarkers

Serum catalase (CAT), superoxide dismutase (SOD), and glutathione peroxidase (GPX) enzyme activities, as well as malondialdehyde (MDA) levels (as a marker of lipid peroxidation), were evaluated colorimetrically using commercially purchased specific diagnostic kits (Bio diagnostic Company, Giza, Egypt).

### 2.9. Gene Transcription

The total RNA was extracted from 100 mg of the hepatic tissues using Trizol reagent (in accordance with the protocol obtained from the manufacturer). The quality and quantity of the RNA were then confirmed by Nanodrop (Uv–Vis spectrophotometer Q5000/Quawell, USA). The complementary DNA (cDNA) was synthesized using the SensiFAST™ cDNA synthesis kit (Bioline, London, UK) (according to the protocol of the manufacturer). The cDNA samples were then stored at −20 °C until use.

Specific primer sequences were used for evaluation of the mRNA expression levels of some immune-related genes such as interleukin 1beta (*IL-1β*), *IL-8*, and interferon-gamma (*INF-γ*), and stress-related genes such as heat shock protein 70 (*HSP70*). Moreover, beta-actin (*β-actin*) was used as a reference gene (Table S2—please see Supplementary Materials).

The SYBR green method was used to quantify the gene transcription folds using an RT-PCR machine (SensiFast SYBR Lo-Rox kit, Bioline, London, UK). The thermal cycle conditions for the reaction were adjusted as follows: 10 min at 95 °C, 40 cycles at 95 °C for 15 s, 30 min at 60 °C, and finally 5 min at 85 °C (61 °C for *IFN-γ*) for 1 min. The transcription folds were standardized to *β-actin* through using the 2^−ΔΔCT^ method [46].

### 2.10. Statistical Analysis

Data were checked for normality and homogeneity using the Levene test. The non-percentage data were directly represented, but the percentage of data was arcsine transformed before analysis. A Two Way-ANOVA in a 2 × 4 factorial design which represents two main factors—two levels of ASP and four levels of salinities—was used to obtain the main effects of ASP, salinity, and the interaction between them. The significant differences were determined at the level of *p* < 0.05. The differences between the calculated means were determined using Tukey’s range test. The statistics were calculated using the SPSS program version 22 (SPSS, v 22.0; SPSS Inc., Chicago, IL, USA).

## 3. Results

The fish efficiently ingested all tested diets, and no mortalities were found in all experimental groups (CTR and ASP) after exposure to different salinity levels for 15 days.

### 3.1. Serum Biochemical Measurements

Two-way ANOVA analysis showed that the effects of salinity levels on the CTR group showed significant increases (*p* < 0.05; Table 1) in GLU, CORT, ALT, and AST levels. These results suggested the occurrence of stress in the exposed fish. Moreover, there were significant decreases in TP, ALB, and GLO values of salinity-exposed fish in the CTR group. The opposite trend was, however, noticed in the ASP-supplemented group, which was exposed to the same salinities, whereas the individual effects of ASP in Nile tilapia exposed to different salinity levels showed a significant decrease in blood GLU, CORT, ALT, and AST values (*p* < 0.05; Table 1), alongside a significant increase in ALB, GLO, and TP values (*p* < 0.05; Table 1) compared to the CTR group exposed to the same salinity levels.

### 3.2. Non-Specific Immune Parameters

Through pairwise comparisons with the CTR group exposed to different salinity levels, it was found that the individual effects of ASP-supplemented diets in Nile tilapia exposed to the same salinities included a significant increase in the serum LYZ activity and phagocytic capacity of blood macrophages (PA % and PI) (*p* < 0.05; Table 2).

### 3.3. Antioxidative Capacity Markers

Compared with the CTR group exposed to different salinities, ASP-supplementation induced significant effects in the antioxidative biomarkers of treated fish (*p* < 0.05). Nile tilapia fed on ASP-supplemented diets and concurrently exposed to different salinities had significantly lowered serum MDA levels alongside elevated serum CAT, SOD, and GPX activities compared with the CTR group exposed to the same salinities (*p* < 0.05; Table 3).

### 3.4. Transcriptomic Profile

**Figure 2:**
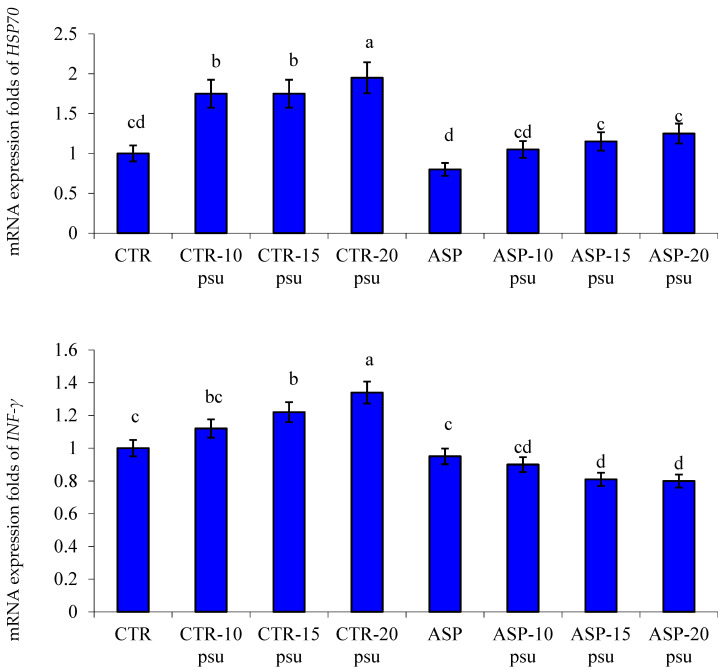
Changes in the mRNA expression folds of hepatic *HSP70* and *INF-γ* genes in Nile tilapia fed a basal diet (CTR) in comparison to those fed on diets supplemented with *Aspergillus oryzae* (ASP) for 12 weeks and then exposed to different salinity levels for 15 days (10, 15, and 20 psu). Bars of each variable assigned by different letters are statistically significant. *p* values of Two-way ANOVA (ASP = 0.022, salinity = 0.034, and ASP × salinity interaction = ˂0.001).

**Figure 3:**
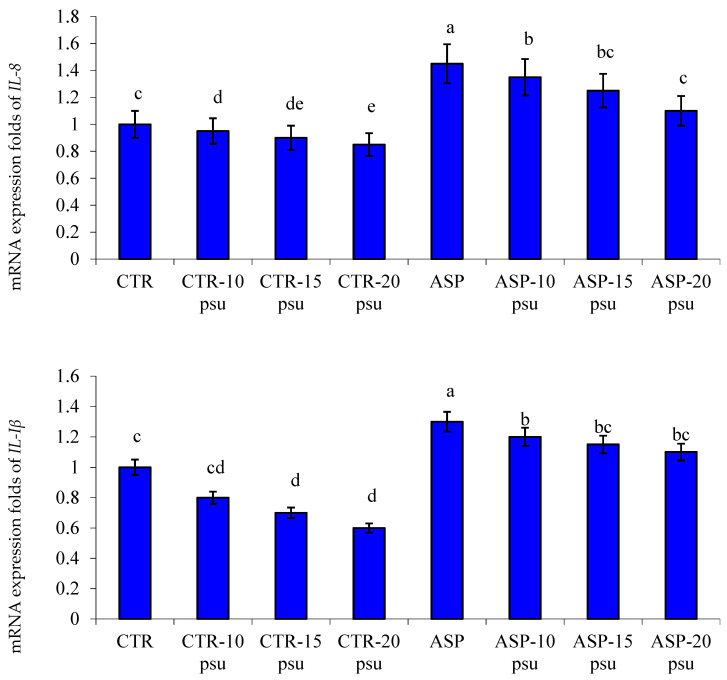
Changes in the mRNA expression folds of hepatic *IL-8* and *IL-1β* genes in Nile tilapia fed a basal diet (CTR) in comparison to those fed on diets supplemented with *Aspergillus oryzae* (ASP) for 12 weeks and then exposed to different salinity levels for 15 days (10, 15, and 20 psu). Bars of each variable assigned by different letters are statistically significant. *p* values of Two-way ANOVA (ASP = 0.022, salinity = 0.034, and ASP × salinity interaction = ˂0.001).

There was significant downregulation of the expression profile of HSP70 and INF-γ genes in hepatic tissues of Nile tilapia fed on ASP-supplemented diets and exposed to different salinity levels compared to those in the CTR group and exposed to the same salinities (*p* < 0.05; Figure 2). Besides, there was significant upregulation of the expression profile of IL-8 and IL-1β genes in the hepatic tissues of Nile tilapia fed an ASP diet and exposed to different salinities than the CTR group exposed to the same salinities (*p* < 0.05; Figure 3).

From the aforementioned findings, the potential capacity of ASP in the improvement of fish immunity, via the upregulation of immune-related genes and the down regulation of stress-related genes, in salinity stress-exposed Nile tilapia can be suggested.

## 4. Discussion

For a long period, the use of probiotics in aquaculture had gained great interest because of their growth-promoting and immune-stimulating characteristics [47,48], and recently, they have been increasingly used to alleviate the health problems induced after the exposure of fish to certain adverse environmental stressors and toxic contaminants [49]. Among the probiotics, *A. oryzae* has attracted the interest of researchers for its beneficial roles in maintaining fish health [11,14].

This study evaluated the roles of dietary supplementation with ASP to mitigate the undesirable influences of salinity stress on exposed Nile tilapia. The tested dose was 1 g ASP per kg diet. This dose was selected based on the previous studies conducted in our laboratory. The results suggest that supplementation with 1 g ASP per kg of diet considerably improved growth, intestinal histomorphometry, and hematological profiles [11], enhanced immunity and disease resistance [12], and fortified antioxidative capacities against hypoxia [13].

### 4.1. Serum Biochemical Indices

High CORT levels indicate exposure of fish to stress [50]. Blood GLU is normally secreted for energy production to diminish the adverse impacts of stress on fish [51]. Serum transaminases (AST and ALT) are declarative of the fish liver functions [52]. Blood protein profiles (TP, ALB, and GLO) are considered as effective indicators of the humoral immunity of fish [53]. There was a significant decrease in blood GLU, CORT, ALT, and AST values, alongside a considerable increase in ALB, GLO, and TP values in the ASP groups exposed to different salinity levels compared to those in the CTR group exposed to the same salinities. The elevation of blood GLU and CORT levels in the CTR group exposed to higher salinities are indicative of the effects of salinity stress [54]. The decrease in CORT and GLU levels in ASP-supplemented groups, and concurrently, exposure to different salinities suggest the beneficial effects of ASP in the relief of the side effects of salinity stress. Our findings were consistent with those obtained by Dawood et al. [13], who demonstrated that dietary ASP noticeably decreased the CORT and GLU levels in Nile tilapia exposed to hypoxia stress. Moreover, dietary supplementation with *Pediococcus acidilactici* considerably lowered the blood GLU and CORT in green terror during hypoxia [55]. Similarly, CORT and GLU levels were significantly decreased in red sea bream fed diets supplemented with *Lactobacillus rhamnosus* and exposed to low salinity stress [33].

The increases in ALT and AST enzyme activities are also indicative of damage, necrosis, and degeneration of hepatic tissues [56]. There were significant decreases in the ALT and AST levels in the serum of Nile tilapia fed on ASP-supplemented diets and exposed to salinity stress as compared with the CTR group exposed to salinity stress. These results were in line with those obtained by Dawood et al. [11]. Furthermore, dietary supplementation with the prebiotic mannan oligosaccharide considerably decreased the ALT and AST levels in red sea bream exposed to low salinity stress [36].

### 4.2. Immune and Antioxidant Measurements

Phagocytic cells are an important component of the cellular immune system in fish, with protective mechanisms against invading pathogens [57]. Moreover, lysozyme is important for lysis of the cell walls of the pathogenic G+ve and G-ve bacteria [43,58]. It has been reported that exposure of Nile tilapia to physical stressors such as heat stress and high stocking densities has led to immune suppression of the exposed fish [2,59]. In the present study, compared to the CTR group exposed to different salinity levels, the effects of ASP-supplemented diets in Nile tilapia exposed to the same salinities showed a significant increase in the serum LYZ activity and phagocytic capacity of the macrophages (PA % and PI). These findings suggest the immune-modulating roles of dietary ASP against the salinity stress-induced decline in the non-specific immunity of exposed Nile tilapia. Several probiotics significantly increased the LYZ activity and phagocytic capacity of fish [7,49,60,61].

Oxidative stress is defined as the state induced through the overproduction of reactive oxygen species combined with the inability of the host body to counteract their side effects, leading to lysis of the cell membrane, disruption of the cellular components, and lipid peroxidation (LPO) [62,63]. It has been reported that oxidative stress occurs after exposure of the living organisms to harmful substances [64,65,66,67]. On the other hand, the CAT, SOD, and GPX enzymes comprise part of the enzymatic antioxidant mechanism to alleviate oxidative stress [68], and MDA is a bioindicator of LPO [69]. In the existing study, Nile tilapia fed on ASP-supplemented diets and exposed to different salinities had significantly lowered serum MDA levels alongside elevated serum CAT, SOD, and GPX activities compared with the CTR group exposed to the same salinities. These findings suggest the potential role of dietary ASP in potentiating the antioxidative capacity in salinity stress-exposed fish.

In line with our findings, Esteban et al. [70] showed that dietary probiotics potentiate the enzymatic antioxidant mechanisms in gilthead seabream (*Sparus aurata*). Dawood et al. [12] illustrated that the synbiotic effects of ASP and beta-glucan significantly potentiate the antioxidant capacity of Nile tilapia. Moreover, Dawood et al. [13] reported a similar substantial increase in SOD, CAT, and GPX activities alongside a decrease in MDA levels in Nile tilapia fed ASP-supplemented diets and exposed to hypoxia stress.

### 4.3. Gene Transcription

Cytokines are signaling molecules generated by immune cells to promote inflammation at the infection site, and consequently, accelerate the arrival of phagocytic cells to engulf and kill the invading pathogenic agents [24,71]. *IL-1β* is a pro-inflammatory cytokine that performs a pivotal role in regulation of the inflammatory and immune processes [72]. *INF-γ* is one of the interferons that play an important role in fish immunity [73,74]. *HSP70* is a stress marker in aquatic animals [25,75,76]. In the present study, it was noted that before and after exposure to the salinity stress, there was a significant upregulation of the mRNA expression values of *IL-8* and *IL-1β* genes alongside downregulation of the mRNA expression values of *HSP70* and *INF-γ* genes in the liver of Nile tilapia fed on ASP-supplemented diets. Xia et al. [77] showed that dietary probiotics considerably increased the expression of *IL-1β* in Nile tilapia. Moreover, the expression of *IL-8* and *IL-1β* was significantly increased in grass carp fed on diets supplemented with probiotics [78].

## 5. Conclusions

The findings of the existing study showed that the exposure to higher salinities induced haemato-biochemical alterations, a decline in the immune parameters, and oxidative stress in the exposed Nile tilapia. Moreover, the dietary probiotic effect of *A. oryzae* was clearly noticed in the improvement of the growth performance and alleviation of the side effects of salinity stress. Taken together, the improvement of serum biochemistry, such as cortisol and glucose values, the enhancement of fish immunity, the modulation of antioxidative capacity, and the transcriptomic profile of the exposed fish were indicative of the beneficial roles of *A. oryzae*-supplemented diets in the alleviation of the side effects of salinity stress in Nile tilapia.

## Figures and Tables

**Table 1:** Serum biochemical measurements of Nile tilapia fed a basal diet (CTR) compared to those fed on diets supplemented with Aspergillus oryzae (ASP) for 12 weeks and then exposed to different salinity levels for 15 days.

Treatment		Serum Biochemical Parameters						
Diets	Salinity Levels	GLU (mg/dL)	CORT (ng/mL)	GLO (g/dL)	ALB (g/dL)	TP (g/dL)	AST (U/L)	ALT (U/L)
CTR	0.0 psu	10.25	54.28	1.46	1.26	2.72	24.11	30.01
	10 psu	10.92	65.73	1.42	1.25	2.67	24.95	35.42
	15 psu	11.15	69.94	1.40	1.24	2.63	25.62	36.69
	20 psu	11.50	70.71	1.37	1.20	2.57	26.15	38.04
ASP	0.0 psu	10.13	41.46	1.62	1.41	3.03	23.97	29.11
	10 psu	10.76	52.18	1.53	1.38	2.91	24.66	30.33
	15 psu	10.88	53.16	1.58	1.29	2.87	24.89	32.70
	20 psu	10.97	50.90	1.58	1.21	2.78	24.99	34.22
SEM		0.05	0.65	0.02	0.01	0.01	0.04	0.11
Two-way ANOVA (*p* values)								
ASP		˂0.001	˂0.001	˂0.001	˂0.001	˂0.001	˂0.001	˂0.001
Salinity		˂0.001	0.031	0.266	˂0.001	˂0.001	˂0.001	˂0.001
ASP × Salinity		0.034	0.012	0.001	˂0.001	0.097	˂0.001	0.001

Data were presented as means ± standard error. GLU: Glucose, CORT: Cortisol, GLO: Globulin, ALB: Albumin, TP: Total protein, AST: Aspartate transaminase, ALT: Alanine transaminase.

**Table 2:** Non-specific immunity parameters of Nile tilapia fed a basal diet (CTR) compared to those fed on diets supplemented with Aspergillus oryzae (ASP) for 12 weeks and exposed to different salinity levels for 15 days.

Treatments		Measurements		
Diets	Salinity Levels	LYZ (Units/mL)	PI	PA (%)
CTR	0.0 psu	8.80	0.77	9.12
	10 psu	8.84	0.83	9.16
	15 psu	8.84	0.88	9.30
	20 psu	8.64	0.81	9.19
ASP	0.0 psu	9.38	1.02	10.20
	10 psu	9.70	1.10	10.16
	15 psu	9.74	1.15	10.21
	20 psu	9.55	1.01	10.05
SEM		0.02	0.01	0.03
Two- way ANOVA (*p* values)				
ASP		˂0.001	˂0.001	˂0.001
Salinity		˂0.001	˂0.001	0.001
ASP × Salinity		0.323	0.001	0.081

Data were presented as means ± standard error. LYZ: Lysozyme activity, PI: Phagocytic index, PA: Phagocytic activity.

**Table 3:** Serum antioxidant biomarkers of Nile tilapia fed a basal diet (CTR) compared to those fed on diets supplemented with Aspergillus oryzae (ASP) for 12 weeks and then exposed to different salinity levels for 15 days.

Treatment		Serum Parameters			
Diet	Salinity	MDA (nmol/mL)	GPX (IU/L)	CAT (IU/L)	SOD (IU/L)
CTR	0.0 psu	10.71	8.28	7.57	6.90
	10 psu	11.16	8.78	7.73	6.94
	15 psu	11.17	8.77	7.70	7.03
	20 psu	11.34	8.70	7.67	6.84
ASP	0.0 psu	8.63	9.62	8.05	7.14
	10 psu	8.82	10.11	8.37	8.20
	15 psu	8.97	10.27	8.48	8.13
	20 psu	9.01	10.28	8.46	8.29
SEM		0.03	0.01	0.02	0.04
Two-way ANOVA (*p* values)					
ASP		˂0.001	˂0.001	˂0.001	˂0.001
Salinity		˂0.001	0.001	0.022	0.937
ASP × Salinity		0.015	˂0.001	˂0.001	0.002

Data were presented as means ± standard error. MDA: Malondialdehyde, GPX: Glutathione peroxidase, CAT: Catalase, SOD: Superoxide dismutase.

## Data Availability

All datasets collected and analyzed during the current study are available from the corresponding author on fair request.

## Supplementary Materials

The following are available online at https://www.mdpi.com/article/10.3390/ani11061621/s1.
