# Supplementary material for: Dietary Aspergillus oryzae Modulates Serum Biochemical Indices, Immune Responses, Oxidative Stress, and Transcription of HSP70 and Cytokine Genes in Nile Tilapia Exposed to Salinity Stress
Source: Animals (Basel). 2021 May 31;11(6):1621. doi: 10.3390/ani11061621 (PMC8228878; doi:10.3390/ani11061621)
Supplement: Supplementary file 1 [file animals-11-01621-s001.zip › animals-1154636-supplementary.pdf]

animals

MDPI

Article

Supplementary Materials: Dietary *Aspergillus oryzae* Modulates Serum biochemical indices, Immune responses, Oxidative stress, and Transcription of *HSP70* and cytokine genes in Nile tilapia exposed to Salinity stress

Mustafa Shukry, Marwa F. Abd El-Kader, Basma M. Hendam, Mahmoud A.O. Dawood, Foad A. Farrag, Salama Mostafa Aboelenin , Mohamed Mohamed Soliman , Hany M.R. Abdel-Latif

Table S1. The ingredients and proximate chemical composition of the basal diet used for Nile tilapia in the current study.

Ingredients	(g/kg) on Dry Weight Basis	Proximate Chemical Analysis	
Fish meal	100	Crude protein (CP) (%)	29.94
Soybean meal	350	Ether extract (EE) (%)	9.98
Yellow corn	160	Crude fiber (CF) (%)	5.55
Wheat flour	150	Total ash (%)	7.24
Wheat bran	150	Nitrogen-free Extract (%) [c]	47.29
Corn oil	15	Gross energy (Kcal/ kg) [d]	4575
Fish oil	15	Protein energy ratio (P/E)	65.44
Starch	50		
Mineral premix [a]	5		
Vitamin premix [b]	5		
Total (g)	1000		

[a] Mineral premix (per kg of premix): Zinc (40 g), Iron (20 g), Copper (2.7 g), Iodine (0.34 g), Manganese (53 g), Selenium (70 mg) and Cobalt (70 mg) and calcium carbonate as carrier up to 1 kg; [b] Vitamin premix (per kg of premix): Vitamin B_1 (700 mg), Vitamin B_2 (3500 mg), Vitamin B_6 (1000 mg), Vitamin B_{12} (7 mg), Vitamin A (8000000 IU), Vitamin D_3 (2000000 IU), Vitamin E (7000 mg), Vitamin K_3 (1500 mg), biotin (50 mg), folic acid (700 mg), nicotinic acid (20000 mg), and pantothenic acid (7000 mg); [c] Nitrogen free extract (calculated by differences) (NFE) = 100- (CP%+ EE%+ CF%+ Ash%)]; [d] Gross energy (GE) value was calculated from the chemical composition as 5.64, 9.44 and 4.11 Kcal/g for protein, lipids and NFE, respectively.

Table S2. The primer sequences of genes used for RT-PCR analysis.

Target mRNA	Primer Sequences (F: Forward, R: Reverse)	NCBI GenBank Accession No.
HSP70	F: 5′-CATCGCCTACGGTCTGGACAA-3′<br>R: 5′-TGCCGTCTTCAATGGTCAGGAT-3′	FJ207463.1
IL-8	F: 5′-TCATTGTCAGCTCCATCGTG-3′<br>R: 5′-CCTGTCCTTTTCAGTGTGGC-3′	NM_001279704.1
IL-1β	F: 5′-CAAGGATGACGACAAGCCAACC-3′<br>R: 5′-AGCGGACAGACATGAGAGTGC-3′	XM_003460625.2
IFN-γ	F: 5′-AGCACAACGTAGCTTTCCCT-3′<br>R: 5′-TAAACAGGGCAAACAGGTCA-3′	XM_003460533.2
β-actin	F: 5′-CCACACAGTGCCCATCTACGA-3′<br>R: 5′-CCACGCTCTGTCAGGATCTTCA-3′	EU887951.1

HSP70: Heat shock protein 70, *IL-8*: Interleukin 8, *IL-1β*: Interleukin 1beta, *IFN-γ*: Interferon gamma, *β-actin*: Beta actin (house-keeping gene).
